# Short-Term Outcomes of Lupus Nephritis: A Single-Center Experience From South India

**DOI:** 10.7759/cureus.91596

**Published:** 2025-09-04

**Authors:** Vijaya Durairaj Kottursamy, Thirumalvalavan Kaliaperumal, Srinivasa Prasad N D, Poongodi Annadurai, Sujith Surendran, Thirumavalavan Subramanian, Murugesan Vellaisamy, Edwin Fernando

**Affiliations:** 1 Nephrology, KAP Viswanatham Government Medical College and Hospital, Trichy, IND; 2 Nephrology, Government Stanley Medical College and Hospital, Chennai, IND

**Keywords:** cyclophosphamide, induction, lupus nephritis, mycophenolate, remission, systemic lupus erythematosus

## Abstract

Background

Systemic lupus erythematosus is a multisystem chronic autoimmune disease that predominantly affects women of reproductive age. Patients with lupus nephritis (LN) are at an increased risk of mortality.

Methodology

In this study, we aimed to compare the efficacy of three different induction regimens, namely, the National Institutes of Health (NIH) regimen, the European Lupus Nephritis Trial (ELNT) regimen, and the mycophenolate mofetil (MMF) regimen, for the treatment of proliferative LN in our study population.

Results

Of the 50 patients, 49 (98%) were women. The mean age, urine protein-creatinine ratio, and serum creatinine of the study participants at the time of diagnosis were 31.96 ± 10.68 years, 2.52 (1.2-4.05) mg/mg, and 1.44 (0.8-1.72) mg/dL, respectively. On renal biopsy, 12 (24%) had class III, 8 (16%) had class III + V, 23 (46%) had class IV, and 7 (14%) had class IV + V LN. Based on factors such as disease activity, patient’s age, risk of infertility, treatment adherence, prior exposure to cyclophosphamide, and patient willingness, 12 (24%) were treated with the NIH regimen, 18 (36%) with the ELNT regimen, and 20 (40%) with oral MMF as induction regimen. At the end of six months, outcomes were assessed. In the NIH group, 83.34% achieved complete remission and 16.67% achieved partial remission. In the ELNT group, 61.1% achieved complete remission, while 38.89% achieved partial remission. In the MMF group, 80% achieved complete remission, 10% achieved partial remission, and 10% did not achieve any remission after six months. No statistically significant difference in response was found between the three induction treatment groups (p = 0.114). The occurrence of intercurrent infections during treatment was 16.67% in the NIH group, 22.2% in the ELNT group, and 20% in the MMF group, without any statistically significant difference in the infection rate between the groups (p = 0.697).

Conclusions

This study demonstrates that the NIH, ELNT, and MMF regimens are equally effective and safe in inducing remission in patients with proliferative LN in our study population.

## Introduction

Systemic lupus erythematosus (SLE) is a multisystem chronic autoimmune disease that predominantly affects women of reproductive age. Lupus nephritis (LN) is one of the most common and serious complications of SLE. The incidence of LN in patients with SLE is 20%-60%, depending on the study population [1]. In men, SLE tends to have a more aggressive course with a higher risk for renal and cardiovascular involvement. LN develops early in the disease course of SLE, generally within the first 6-36 months, and is likely the initial presentation in 35% of patients. In SLE, patients with LN are associated with significantly higher mortality compared to those without LN. Kidney disease progresses to death directly in 5%-25% of patients with proliferative LN within five years. Among patients with LN, 10%-30% of patients progress to kidney failure, requiring renal replacement therapy (RRT) [2]. Patients with proliferative forms of LN (class III, IV, or III/IV + V) are at a higher risk of requiring RRT. Achieving a complete clinical response to the treatment is critical for preserving long-term kidney health. Patients with a complete clinical response had a 92% kidney survival rate at 10 years compared to 43% in partial responders and 13% in non-responders [2]. To achieve a complete clinical response, patients require toxic induction and maintenance immunosuppressive therapy. Cyclophosphamide, which has been used traditionally as an induction regimen in the treatment of LN, is associated with significant short-term and long-term dose-dependent adverse effects. Efforts to reduce the toxicity of cyclophosphamide have focused on modifying its route of administration, dosage, or exploring safe alternative medications such as mycophenolate mofetil (MMF). Studies indicate that the response to induction regimens is not uniform, showing variations among different population groups. The present study aimed to compare the efficacy, safety, and response of three different regimens, namely, the National Institutes of Health (NIH) regimen, the European Lupus Nephritis Trial (ELNT) regimen, and the MMF regimen, in proliferative LN in our study population.

This article was previously presented as a meeting abstract at the ISN World Congress of Nephrology (WCN) 2025, 6-9 February 2025 (available online 27 January 2025).

## Materials and methods

This prospective, observational study was conducted among patients attending the Nephrology Department of our institute between October 2023 and December 2024. Patients of all age groups and either sex who were diagnosed to have SLE as per the 2019 American College of Rheumatology/European League Against Rheumatism (ACR/EULAR) criteria and biopsy-proven class III, IV, V, III + V, or IV + V LN based on the International Society of Nephrology/Renal Pathology Society (ISN/RPS) 2018 classification were included. Written informed consent was taken from all participants. This study was approved by the Institutional Ethics Committee of Government Stanley Medical College and Hospital, Chennai (registration number: ECR/131/Inst/TN/2013/RR-22).

**Figure 1:**
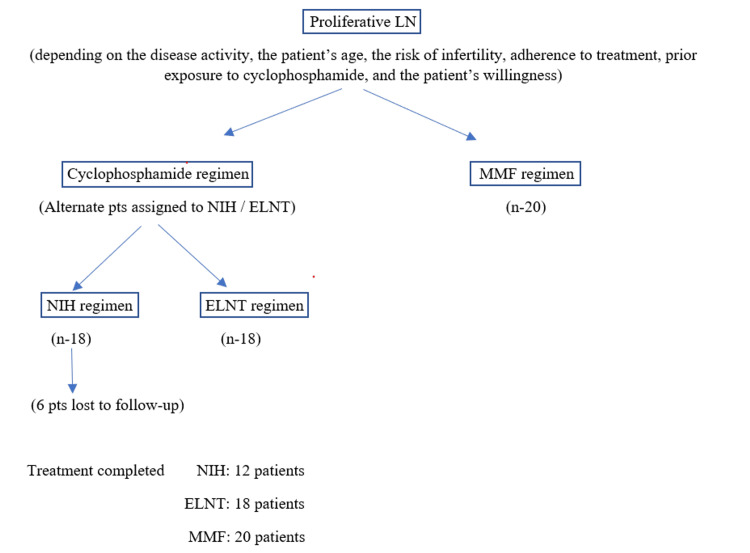
Study protocol. LN = lupus nephritis; NIH = the National Institutes of Health regimen; ELNT = the European Lupus Nephritis Trial regimen; MMF = the mycophenolate mofetil regimen

As per the inclusion criteria, patients diagnosed with proliferative LN were started on one of the three regimens, namely, the NIH regimen, the ELNT regimen, or the MMF regimen, depending on the disease activity, patient age, the risk of infertility, treatment adherence, prior exposure to cyclophosphamide, and patient willingness. Patients diagnosed with proliferative LN and decided to start therapy with cyclophosphamide were assigned to receive either the NIH or the ELNT regimen, with alternate patients assigned to each group. Six patients in the NIH treatment group were lost to follow-up and did not complete the treatment. The 50 patients described in the results completed the treatment and were on regular follow-up (Figure 1).

Patients were followed up every two weeks through week 24. At the start of the study, baseline investigations, including urine examination, complete blood count, renal function tests, serum albumin, urine protein-creatinine ratio (uPCR), antinuclear antibodies, anti-double-stranded deoxyribonuclease antibodies, and complement levels, were performed. Complete blood count, renal function tests, and uPCR were examined at each follow-up.

Response to therapy was defined as per the 2021 Kidney Disease Improving Global Outcomes (KDIGO) criteria for LN. Complete response was defined as a reduction in proteinuria to less than 0.5 g/g (50 mg/mmol), measured as the PCR from a 24-hour urine collection, and stabilization or improvement in kidney function (±10%-15% of baseline) within 6-12 months of starting therapy, but it could take more than 12 months. Partial response was defined as a reduction in proteinuria by at least 50%, to less than 3 g/g (300 mg/mmol), measured as the PCR from a 24-hour urine collection, and stabilization or improvement in kidney function (±10% to 15% of baseline), within 6-12 months of starting therapy. No response was defined as the failure to achieve a partial or complete response within 6-12 months of starting the treatment. Renal remission rates were assessed at the end of six months. Descriptive analysis was performed for baseline characteristics of the study population, the three different induction regimen groups, and the response to different induction regimens.

In the NIH regimen, patients were treated with intravenous (IV) cyclophosphamide infusion at a monthly dose of 0.5-1 g/m² for six months. In the ELNT regimen, patients were treated with IV cyclophosphamide infusion at a fixed dose of 500 mg every two weeks for three months. Patients who decided to be treated with MMF were initially given 500 mg twice a day, and the dose was increased every two weeks to achieve a target dose of 2 g/day. All patients received 250-500 mg/day of IV methylprednisolone as appropriate, depending on disease severity and rate of progression for the initial three days, followed by oral prednisolone at a dose of 0.5-1.0 mg/kg/day (maximum of 80 mg/day) and subsequent tapering to a maintenance dose of 5.0-7.5 mg/day. All patients were treated with hydroxychloroquine at a dose of 5 mg/kg/day. Patients were started on angiotensin-converting enzyme inhibitors/angiotensin receptor blockers and statins as per indication.

The data were entered into Microsoft Excel and analyzed using SPSS version 21 (IBM Corp., Armonk, NY, USA). Categorical variables were represented using frequencies and percentages. Continuous variables that followed a normal distribution were described as mean ± standard deviation, and those that did not follow a normal distribution were depicted as median and interquartile range. Appropriate statistical tests, including chi-square and t-tests, were applied for bivariate analysis. A P-value of less than 0.05 was considered statistically significant.

## Results

In total, 50 patients were included in the study. Of them, 49 (98%) were women, and one (2%) was a man. The mean age of the study participants was 31.96 ± 10.68 years. The mean uPCR of the study population was 2.52 (1.2-4.05) mg/mg. Among the participants, 42 (84%) had active sediments (the presence of red blood cells in urine in the absence of infection) in urine at presentation. The mean serum creatinine of the study population was 1.44 (0.8-1.72) mg/dL, and 15 (30%) patients had hypertension at presentation (Table 1).

**Table 1:** Basic characteristics of the study participants. PCR = protein-creatinine ratio; ISN/RPS = International Society of Nephrology/Renal Pathology Society; IQR = interquartile range

Characteristics	n (%) or mean ± SD or median (IQR)
N	50
Mean age (years)	31.96 ± 10.68
Female:Male	49:1
Proteinuria (PCR)	2.52 (1.20–4.05)
Active urinary sediments	42 (84%)
Serum creatinine (mg/dL)	1.44 (0.8–1.72)
Hypertension	15 (30%)
Renal biopsy (ISN/RPS) 2018 classification	
Class III	12 (24%)
Class III and V	8 (16%)
Class IV	23 (46%)
Class IV and V	7 (14%)

On renal biopsy, 12 (24%), 8 (16%), 23 (46%), and 7 (14%) participants had class III, class III + V, class IV, and class IV + V LN, respectively. Crescentic glomerulonephritis was present in one (2%) patient.

Depending on the disease activity, patients’ age, risk of infertility, adherence to treatment, prior exposure to cyclophosphamide for other indications, and patients’ willingness, 12 (24%), 18 (36%), and 20 (40%) patients were started on the NIH regimen, ELNT regimen, and oral MMF as the induction regimen, respectively. The outcome was assessed at the end of six months.

There was no significant difference in the treatment group’s mean age, uPCR, serum creatinine, complement levels, class of LN, activity index, and chronicity index in renal biopsy (chi-square test/independent t-test) (Table 2).

**Table 2:** Basic characteristics of different induction treatment groups. *: P-value <0.05 is considered significant. NIH = the National Institutes of Health regimen; ELNT = the European Lupus Nephritis Trial regimen; MMF = the mycophenolate mofetil regimen; PCR = protein-creatinine ratio; ANOVA = analysis of variance

Characteristic	NIH (n = 12)	ELNT (n = 18)	MMF (n = 20)	Statistical test	Degree of freedom	Statistical value (t, χ², F, or H)	P-value*
Mean age (years)	36.17 ± 7.77	32.94 ± 11.10	28.55 ± 11.18	ANOVA	2	F ≈ 0.50	0.611
Female:Male	12:0	17:1	20:0	Fisher’s exact test	2	FE ≈ 1.773	0.600
Proteinuria (PCR)	3.70 (1.90–4.98)	2.70 (1.87–5.62)	1.40 (0.90–2.64)	Kruskal–Wallis test	2	H ≈ 2.53	0.280
Active urinary sediments	11 (91.7%)	15 (83.3%)	16 (80%)	Chi-square test	2	-	
Serum creatinine (mg/dL)	1.15 (0.82–2.67)	1.10 (0.87–2.20)	0.95 (0.80–1.18)	Kruskal–Wallis test	2	H ≈ 1.27	0.529
Complement C3 level	56.53 ± 26.99	60.15 ± 24.80	59.5 (33.2–108.0)	ANOVA	2	F ≈ 0.93	0.401
Complement C4 level	8.40 (4.30–15.07)	8.50 (4.30–11.70)	7.6 (2.92–15.5)	Kruskal–Wallis test	2	H ≈ 0.97	0.615
Hypertension	4 (33.3%)	6 (33.4%)	5 (25%)	Chi-square test	2	-	
Activity index (/24)	8.0 ± 3.8	7.5 ± 3.75	3.0 ± 2.8	Kruskal–Wallis	2	H ≈ 4.82	0.089
Chronicity index (/12)	1.0 (0–2.0)	0.83 (0–1)	0.0 (0–1.75)	Kruskal–Wallis test	2	H ≈ 2.63	0.269
Renal biopsy class							
Class III	3	4	5	Fisher’s exact test	6	FE ≈ 10.79	0.554
Class III and V	2	2	4				
Class IV	5	11	7				
Class IV and V	2	1	4				

The outcomes were measured as no remission, partial remission, and complete remission based on the KDIGO criteria. In the NIH induction treatment group, out of 12 patients, 10 (83.34%) achieved complete remission, and two (16.67%) achieved partial remission at the end of six months. In the ELNT induction treatment group, out of 18 patients, 11 (61.1%) achieved complete remission, and seven (38.89%) achieved partial remission at the end of six months. In the MMF induction treatment group, out of 20 patients, 16 (80%) achieved complete remission, two (10%) achieved partial remission, and two (10%) did not achieve partial or complete remission at the end of six months. There was no statistically significant difference in response between the three induction treatment groups (p = 0.134; Fisher’s exact test) (Table 3).

**Table 3:** Induction regimen and responses based on the KDIGO criteria. ^#^: Fisher’s exact test; *: P-value <0.05 is considered significant. NIH = the National Institutes of Health regimen; ELNT = the European Lupus Nephritis Trial regimen; MMF = the mycophenolate mofetil regimen; KDIGO = Kidney Disease Improving Global Outcomes

Regimen/Response	No response	Partial remission	Complete remission	Test value^#^	P-Value*
NIH	0	2	10	6.032	0.134
ELNT	0	7	11		
MMF	2	2	16		

In the NIH, ELNT, and MMF induction groups, two (16.67%), four (22.2%), and four (20%) patients had intercurrent infections during the treatment, respectively (Table 4). In the NIH induction group, three (25%) patients experienced transient amenorrhea. In the NIH and ELNT induction group, five (41.6%) and seven (38.8%) patients developed leucopenia, respectively. The leucopenia episodes were transient and did not warrant dose modification of immunosuppressive therapy or rescue treatment with granulocyte-macrophage colony-stimulating factor.

**Table 4:** Occurrence of infections in the three induction treatment groups. NIH = the National Institutes of Health regimen; ELNT = the European Lupus Nephritis Trial regimen; MMF = the mycophenolate mofetil regimen; UTI = urinary tract infection; LRTI = lower respiratory tract infection

Infection type	NIH	ELNT	MMF
Dermatophytosis	1	–	–
Mastitis	–	–	1
Otitis media	–	1	–
Herpes zoster	–	1	1
UTI	–	1	1
LRTI	1	1	1
Total (n, %)	2 (16.67%)	4 (22.2%)	4 (20%)

## Discussion

In this study, we compared three types of induction treatment (NIH regimen, ELNT regimen, and oral MMF regimen) for the management of severe proliferative forms of LN. We observed that the treatment outcome and adverse event rates were comparable between the treatment groups.

Initial research into treating proliferative LN highlighted the effectiveness of cyclophosphamide. In the NIH trial (1996), Gourley et al. observed that monthly bolus therapy with cyclophosphamide (0.5-1.0 g/m² body surface area (BSA)) was more effective than monthly bolus therapy with methylprednisolone (1 g/m² BSA) in proliferative LN. When both were combined, a trend toward greater efficacy was observed [3].

However, concerns about cyclophosphamide’s side effects led to efforts to reduce its dosage and explore alternative treatments. The 2002 ELNT study by Houssiau et al. compared different cyclophosphamide dosing regimens (six fortnightly 500 mg fixed doses vs. monthly high-dose injections) in predominantly White patients with proliferative LN. The study found no significant differences in outcomes or adverse events between high- and low-dose IV cyclophosphamide [4].

Subsequent studies began exploring MMF as an alternative. Chan et al. (2000) demonstrated that a combination of MMF and prednisolone was as effective as oral cyclophosphamide and prednisolone for induction treatment of diffuse proliferative LN in a Chinese population [5]. Further supporting this, Hu et al. (2002) concluded that MMF was even more effective than IV pulse cyclophosphamide as an induction agent in Chinese patients with diffuse proliferative LN [6].

The effectiveness and safety of MMF were further explored in several key studies. In 2005, a 24-week randomized trial by Ginzler et al. in the United States found that oral MMF was more effective than monthly IV cyclophosphamide for inducing remission in patients with active LN. This study also noted a favorable safety profile for MMF [7].

However, a broader international perspective emerged from the 2009 Aspreva Lupus Management Study (ALMS). This 24-week induction study, conducted across 20 countries, compared MMF (at a target dosage of 3 g/day) with IV cyclophosphamide (0.5-1.0 g/m² in monthly pulses) in patients with active LN. The ALMS trial concluded there was no significant difference in response rates or adverse events between the MMF and IV cyclophosphamide groups [8].

In 2015, Rathi et al. investigated the efficacy of low-dose IV cyclophosphamide versus oral MMF in active LN patients within a North Indian population. Their findings indicated that low-dose IV cyclophosphamide was as effective as oral MMF for inducing remission in patients with moderately severe LN; notably, those with severe and crescentic LN were excluded from this study [9].

Recently, a comprehensive comparison by Sahay et al. assessed the efficacy and safety of three prominent treatment protocols for proliferative LN, i.e., the ELNT, NIH, and MMF regimens. They concluded that all three regimens demonstrated comparable effectiveness. Furthermore, their analysis highlighted that cyclophosphamide-based regimens remain a cost-effective option in resource-limited settings [10].

Given that responses to induction regimens vary across different population groups, it was crucial to understand how the local population responded to various treatments. This knowledge is essential for achieving complete remission and, consequently, preserving long-term kidney health.

In our study, 83.34% of patients achieved complete remission and 16.67% achieved partial remission in the NIH regimen treatment group; 61.1% achieved complete remission and 38.89% achieved partial remission in the ELNT regimen treatment group; and 80% achieved complete remission and 10% achieved partial remission in the MMF regimen treatment group. Overall, 10% of patients did not achieve remission in the MMF group. The response outcome was not statistically different between the three treatment groups (p = 0.134; Fisher’s exact test).

The MMF induction group exhibited a mean proteinuria (PCR) of 1.40 (0.90-2.64) and a mean activity index of 3.0 (2.0-5.8). Although not statistically different from the other groups, these values were relatively low. This could have influenced the outcome in the MMF group.

The incidence of infection in our study was 16.67%, 22.2%, and 20% in NIH, ELNT, and MMF induction groups, respectively. Our study findings are consistent with those of previous studies in other Indian populations.

Our study population experienced a more favorable response to induction treatment. This may be attributable to the earlier diagnosis, referral, and treatment, as evidenced by lower chronicity indexes in renal biopsy.

This study’s limitation is that it was a single-center, observational study conducted among a single ethnic group. Its shorter duration, small sample size, and restriction to induction therapy alone are other limitations. However, all patients were being followed regularly as outpatients.

## Conclusions

The primary goal of immunosuppressive treatment in LN is to achieve remission, thereby improving patient survival and preserving renal function. The significant short-term and long-term dose-dependent adverse effects of traditionally used cyclophosphamide necessitate the use of safer induction treatments such as MMF. To optimize treatment protocols and improve renal outcomes for LN patients, it is vital to account for how specific geographic populations respond to various induction regimens. The present study has demonstrated that in patients with proliferative LN, the NIH regimen, the ELNT regimen, and the MMF regimen are equally effective with similar safety profiles in inducing remission in our study population. Although the mean proteinuria and activity index were low in the MMF group, this observation lacked statistical significance. Consequently, MMF presents a viable alternative to cyclophosphamide for patients with proliferative LN, particularly favoring younger patients and those concerned about infertility. A larger, multicenter trial with a longer follow-up period is required to provide more robust evidence and to assess long-term outcomes.
